# Study on Temper Embrittlement and Hydrogen Embrittlement of a Hydrogenation Reactor by Small Punch Test

**DOI:** 10.3390/ma10060671

**Published:** 2017-06-19

**Authors:** Kaishu Guan, Jerzy A. Szpunar, Karel Matocha, Duwei Wang

**Affiliations:** 1 School of Mechanical and Power Engineering, East China University of Science and Technology, Shanghai 200237, China; zzuwangduwei@163.com; 2 Advanced Materials for Clean Energy, Department of Mechanical Engineering, University of Saskatchewan, Saskatoon, SK S7N 5A9, Canada; 3 Department of Material Engineering, Faculty of Metallurgy and Materials Engineering, VŠB—Technical University of Ostrava, 17. Listopadu 2172/15, 708 00 Ostrava, Czech Republic; matocha.karel@email.cz

**Keywords:** 3Cr1Mo1/4V steel, small punch test, energy transition temperature, temper embrittlement, hydrogen embrittlement

## Abstract

The study on temper embrittlement and hydrogen embrittlement of a test block from a 3Cr1Mo1/4V hydrogenation reactor after ten years of service was carried out by small punch test (SPT) at different temperatures. The SPT fracture energy *E_sp_* (derived from integrating the load-displacement curve) divided by the maximum load (*F_m_*) of SPT was used to fit the *E_sp_*/*F_m_* versus-temperature curve to determine the energy transition temperature (*T_sp_*) which corresponded to the ductile-brittle transition temperature of the Charpy impact test. The results indicated that the ratio of *E_sp_*/*F_m_* could better represent the energy of transition in SPT compared with *E_sp_*. The ductile-to-brittle transition temperature of the four different types of materials was measured using the hydrogen charging test by SPT. These four types of materials included the base metal and the weld metal in the as-received state, and the base metal and the weld metal in the de-embrittled state. The results showed that there was a degree of temper embrittlement in the base metal and the weld metal after ten years of service at 390 °C. The specimens became slightly more brittle but this was not obvious after hydrogen charging. Because the toughness of the material of the hydrogenation reactor was very good, the flat samples of SPT could not characterize the energy transition temperature within the liquid nitrogen temperature. Additionally, there was no synergetic effect of temper embrittlement and hydrogen embrittlement found in 3Cr1Mo1/4V steel.

## 1. Introduction

Hydrogenation reactors have been widely used in the petrochemical industry as core equipment of hydrogenation technology. After running in a hydrogen-rich environment at relatively high temperatures (in the range of 350–480 °C) and at high pressures and hydrogen partial pressures (in the range of 15–200 bars) for a long period of time, the mechanical properties of the wall of the reactor will degrade due to temper embrittlement (TE) and/or hydrogen embrittlement (HE) [1].

Experience in recent years has shown that the development of temper embrittlement sometimes interferes with the normal operation of relevant components at elevated temperatures and accelerates the development of hydrogen brittleness [2]. Zhang X.’s study showed [3] that the higher the temper embrittlement is, the higher the degree of temper embrittlement induced by hydrogen.

Present studies have implied that temper embrittlement of Cr-Mo steels is linked to the co-segregation of impurities at prior austenitic grain boundaries and the impurities lead to grain boundary embrittlement, and subsequently become a risk of intergranular fracture [1]. Phosphorus and tin are the primary impurity elements that contribute to embrittlement in low alloy commercial steels. Manganese and silicon are known to intensify the effects of impurities. As a result the combined parameters known as the Watanab’s *J*-*factor* and Bruscato *X*-*bar* have been reported, namely [4]:

*J*-*factor* = (Si + Mn) (P + Sn) × 10^4^ [%] (1)

*X*-*bar* = (10P + 5Sb + 4Sn + As)/100 [ppm] (2)

The Watanab’s *J-factor* is used for base materials, and the Bruscato *X-bar* factor is used for weld metals. They are computed from elemental concentrations, expressed in weight percent in *J-factor* and ppm in *X-bar*, respectively. The higher the *J-factor* and *X-bar* is, the higher the degree of TE.

This kind of TE is revisable and can be eliminated by applying a de-embrittlement at 600–650 °C heat treatment [5].

The extent of TE is measured by the shift of the Charpy V-notch (CVN) transition to higher temperatures. This ductile-brittle transition temperature is usually defined by the temperature for which the average impact energy equals 54 J (vTr54 or TK_54*J*_) for hydrogenation reactor materials [1].

For on-site equipment, sampling for standard tensile, or the Charpy impact and fracture toughness test is limited because of the structural integrity requirement. To overcome the restriction caused by the limited availability of materials and the structural integrity of the components being investigated, a quasi-nondestructive testing technique named the small punch test (SPT) was developed [6]. This technique has been used to study the creep related properties, fracture toughness, strength, and fracture appearance transition temperature (FATT) [7,8,9].

The SP fracture energy *E_sp_* is defined by the area under the SPT load-displacement curve up to the displacement at the load drop after the maximum load: $F_{f}=0.8F_{m}$. The energy transition temperature *T_sp_* is assigned at the curve where the energy is equal to the average energy of the upper and low shelf, or half the value of the upper shelf when the lower shelf is absent. It is believed that there is a certain relationship between *T_sp_* and vTr54, or FATT. FATT is calculated from the SP test results using an empirical correlation between *T_sp_*, determined from the temperature dependence of the fracture energy *E_sp_*, and FATT determined from the results of the Charpy V-notch impact tests. Empirical correlations are found based on the fact that steels exhibiting standard Charpy V-notch impact ductile-brittle transition behavior also show this behavior during a SPT, but are usually shifted to a lower temperature [10].

G.R. Prescott [11] proposed a number of studies on 2.25Cr1Mo steel which indicated that the FATT was related to the vTr54 by the following expression, expressed as:

(3) FATT=vTr54+7 K

Many experiments already demonstrated that *T_sp_* is associated with FATT and has an empirical correlation. R Hurst [12] proposed the formula for *T_sp_* and FATT, expressed as:

(4) $$\mathrm{FATT}=1.58T_{sp}+111.9K\;\left(R^{2}=0.89\right)$$

Generally, a 54 *J* impact energy is observed in the range from −80 °C to −30 °C for modern hydrogenation reactor materials, whilst the *T_sp_* is apparently from −217 to −186 °C.

The TE and/or HE of 2.25Cr1Mo/2.25Cr1MoV steels have been widely investigated [1]. However, studies on 3Cr1Mo1/4V steel are seldom reported. In order to study the feasibility of evaluating temper embrittlement and the susceptibility to hydrogen embrittlement by the SPT, the specimens sampled from a 3Cr1Mo1/4V steel test block which was serviced in the reactor for ten years, were tested with SPT at low temperatures. The measured SPT fracture energy divided by the maximum load (*E_sp_/F_m_*) was introduced to obtain *T_sp_*. The increment of *T_sp_* between the as-received state and the de-embrittled state of the material was compared with the Charpy test results.

## 2. Sampling and Testing

### 2.1. Sampling

Testing materials were cut from a block that was exposed to a typical service environment for 10 years in the reactor. The reactor’s operating temperature and pressure were 390 °C and 16.4 MPa. The medium in service was mainly gasoline, however H_2_ and H_2_S were also used. The wall of the reactor was forged with the thickness of 216 mm. The forging was at 960 °C quenched with 690 °C normalized heat treatment, followed by maximum post weld heat treatment. The results of the mechanical properties measurements are listed in Table 1. The chemical composition is presented in Table 2.

Based on the design requirements, the coefficient *J* should be below 100% and *X-bar* should be less than 15 ppm for safe operation. For this test block material, according to Equations (1) and (2), the coefficient *J* is 81.42% and *X-bar* is 11.72 ppm.

The results show that the investigated material could meet this requirement which means that it would not be badly embrittled. The as-received materials were cut from this test block. The loss of embrittlement was examined after the materials were kept for two hours at 650 °C and then air cooled to room temperature.

### 2.2. SPT and Charpy Impact Testing

SPT samples are disks with a diameter of 10 mm and thickness of 0.5 ± 0.005 mm, as recommended by GB/T29456 [13]. The cross-head speed is 1.5 mm/min. The specific SPT jig and experiment process were shown in our previous publication [14]. Charpy impact test specimens were sampled according to GB/T 229-2007 [15] and GB/T 2650-2008 [16] from the base and weld materials.

Temperature is the most important parameter for determining *T_sp_*. Several temperature points from −196 °C to 25 °C were chosen. Liquid nitrogen and a cooling system including a thermal insulation box, a temperature controller, and two high precision thermal resistances were used to record the cooling process to the required temperature. The specimen should be maintained at the testing temperature for 5 min with fluctuations less than ±1 °C. Two or three samples were tested under the same condition to ensure the reliability of the results.

### 2.3. Hydrogen Charging Test

Hydrogen charging was carried out using a cathodic charging cell made of a power supply, a platinum anode, and the tested sample as the cathode. The working electrode was cathodically charged at a constant current of 0.04 A for 1 h at room temperature in a solution containing 0.5 mol/L H_2_SO_4_ with 0.25 g/L As_2_O_3_ used to promote the rate of hydrogen uptake in the material [17]. The SPT was carried out immediately after the hydrogen charging process. The glycerin method was used to measure the hydrogen concentration, which is similar to the mercury method [18]. The hydrogen concentration was found to be approximately 1.47 ppm.

## 3. Results

### 3.1. Load-Displacement Curve of SPT in Different Temperatures

**Figure 1:**
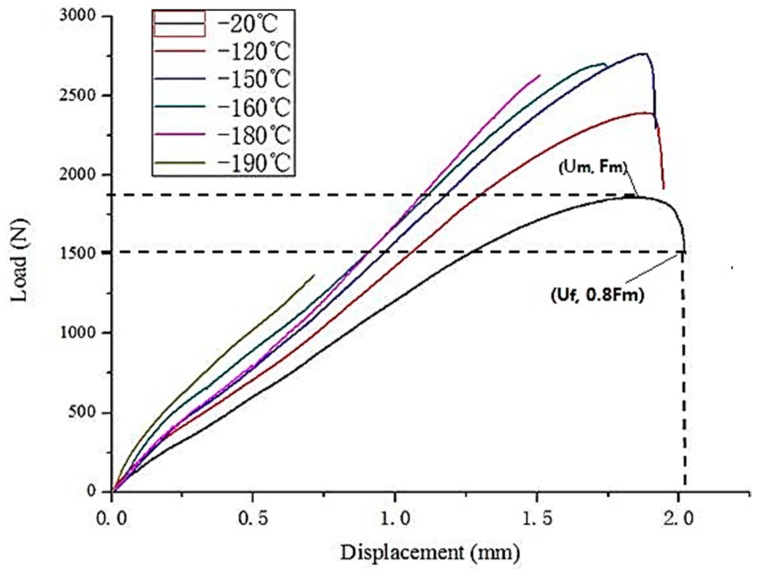
Load-Displacement Curve at different temperatures of the weld metal.

Figure 1 shows the load-displacement curves measured at different temperatures for the weld in the as-received state. From Figure 1, as the temperature decreases from −20 °C to −150 °C, the maximum load, *F_m_*, increases, whilst the displacement corresponding to the fracture at 20% load drop, *U_f_*, decreases slightly.

The shape of the load deflection curve of the investigated specimens can be divided into four typical deformation regimes. There is the elastic stage, the plastic bending regime, the membrane stretching regime, and the plastic instability regime. The curves obtained for temperatures between −20 and −150 °C show all the above stages, with the maximum load from 1700 to 2700 N, and the maximum displacement drops from 2 to 1.8 mm before the break. That means that the strength of the specimens firstly increases and then the plasticity decreases as the temperature decreases from −20 to −150 °C. However for the curves of the remaining specimens, the maximum load and the deflection illustrated in Figure 1 decreased abruptly and the curves show only the first three deformation regimes due to embrittlement in temperatures below −150 °C, i.e., early crack initiation and rapid crack growth during the membrane stretching stage.

The results of *F_m_* and *U_f_* at different temperatures of the weld metal are shown in Table 3. Generally, with the temperature declining, the *F_m_* first increases and then decreases whilst the *U_f_* always decreases.

### 3.2. Energy Transition Temperature of SPT

The fracture energy of the SPT specimen which is abbreviated as *E_sp_*, is commonly derived from integrating the load-displacement curve along the *x*-axis. Finarelli et al. [19] studied SPT specimen deformation and the crack initiation process with a special device. They found that the crack was formed at the peak load point, and the crack decreases the load bearing capacity. It is obvious that toughness is an indicator of strength and plasticity, so even if the specimen could not bear a larger load with the existing crack, it can absorb more energy before it fails.

**Figure 2:**
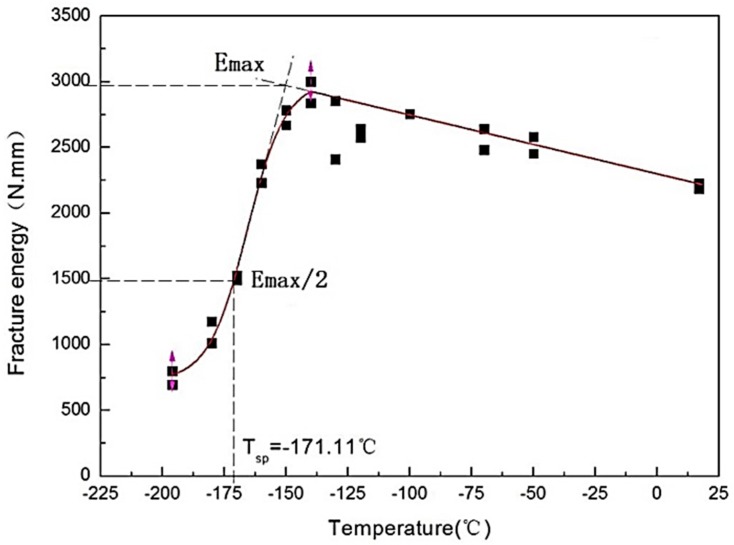
Fitting Energy-Temperature Curve and *T_sp_* of the as-received weld metal.

**Figure 3:**
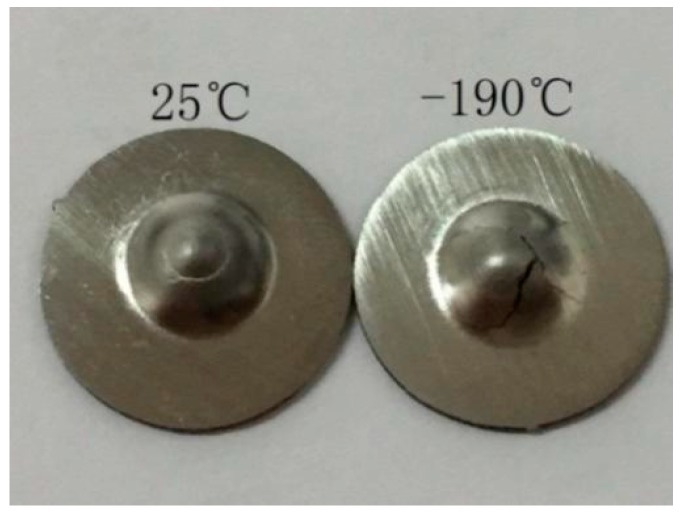
Macrophotograph of the fracture sample at 25 °C and −190 °C.

However, the obtained results show that it is unreasonable to evaluate the energy transition temperature from the energy-temperature curve corresponding to a change in the ductile-to-brittle failure, as seen in Figure 2. Firstly, the increase of the fracture energy from the ambient temperature to nearly −150 °C cannot be explained, in spite of the fact that the specimens are ductile. Secondly, the two specimens can have the same level of fracture energy at totally different temperatures. Figure 3 shows the macro photograph of the fractured samples at 25 °C and at −190 °C. They apparently have significant differences in the morphology of the failure and crack propagation path. The former is ductile fracture and the latter is brittle fracture. However, both specimens have very similar energy values, 2.055 *J* and 2.171 *J*, respectively. Thirdly, it is difficult to determine the upper shelf because the *E_sp_* drops in the upper shelf with increasing temperature. The energy transition temperature *T_sp_* is defined at the curve where the energy is equal to 50% of the maximum energy *E_max_* in Figure 2.

**Figure 4:**
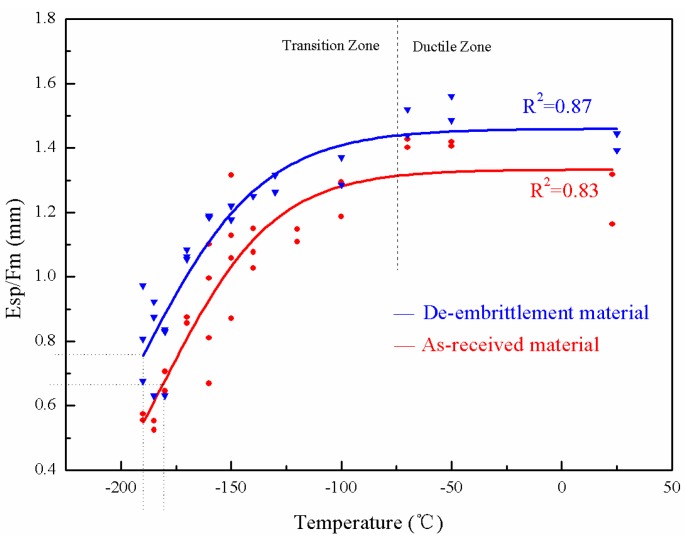
*T-E_sp_*/*F_m_* curve of the base metal.

**Figure 5:**
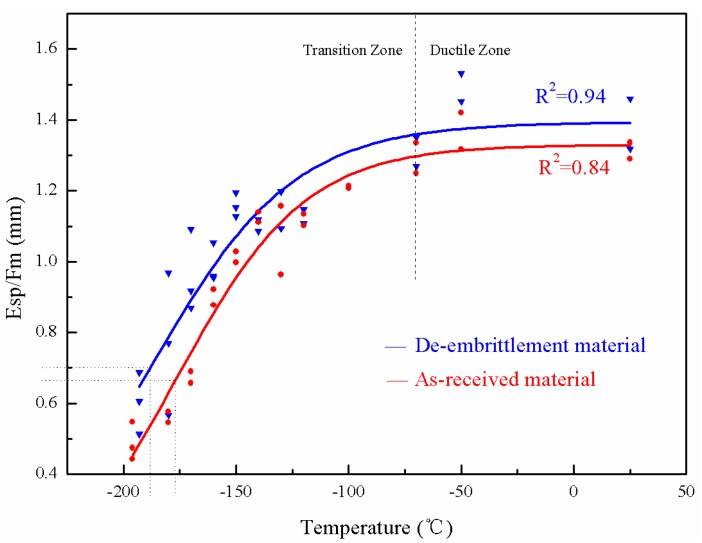
*T-E_sp_*/*F_m_* curve of the weld metal.

When dealing with the low temperature SPT data by fitting the Boltzmann function, *E_sp_* divided by *F_m_* could better represent the energy transition of the materials. The value of *E_sp_*/*F_m_*, being nearly constant above a certain temperature, has much in common with that of FATT or the impact energy. The curves of the de-embrittled material are slightly above the curves of the as-received material, as illustrated in Figure 4 and Figure 5. In Figure 2, Figure 4 and Figure 5, the lower shelf is absent because it is lower than the liquid nitrogen temperature. *T_sp_* is assigned at the curve where the energy is equal to 50% of the upper shelf, instead of the average of the upper and lower shelf.

### 3.3. Temper Embrittlement in the Base Metal and the Weld Metal

There is an offset between the as-received and the de-embrittled material curves for both the base and the weld metals, as shown in Figure 4 and Figure 5. Compared with the as-received material test results, the de-embrittlement curve shifts to the upper-left, indicating that the transition temperature moves towards lower values. Moreover, the average value of *E_sp_*/*F_m_* of the de-embrittled samples at each temperature is higher than that of the as-received specimen.

Table 4 shows *T_sp_* and vTr54 which come from Charpy impact test.

According to Figure 4 and Figure 5, the data in Table 4 show the specific values of *T_sp_* for the base metal and the weld metal in the de-embrittled and as-received material before hydrogen charging. Compared with the data in Table 4, the *T_sp_* of the base metal and the weld metal decreased by 8.9 °C and 11.2 °C, respectively, in the de-embrittled state as opposed to the as-received state before hydrogen charging. The vTr54 of the base metal and the weld metal respectively decreased by 10.7 °C and 13.7 °C in the de-embrittled state as opposed to the as-received state. Both results indicated that there is little temper embrittlement in the base metal and the weld metal of the 3Cr1Mo1/4V hydrogenation reactor after running for ten years.

### 3.4. T_sp_: Hydrogen Embrittlement in the As-Received and Brittle State of the Weld Metal

**Figure 6:**
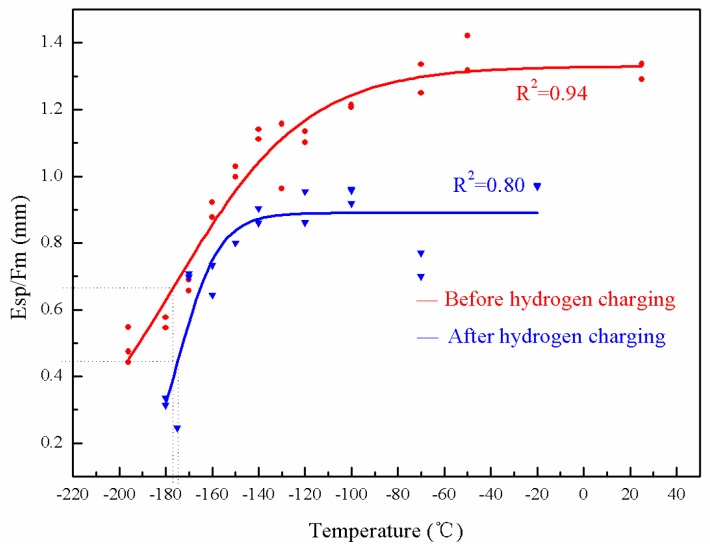
*T-E_sp_*/*F_m_* curve of the weld metal in the as-received state.

**Figure 7:**
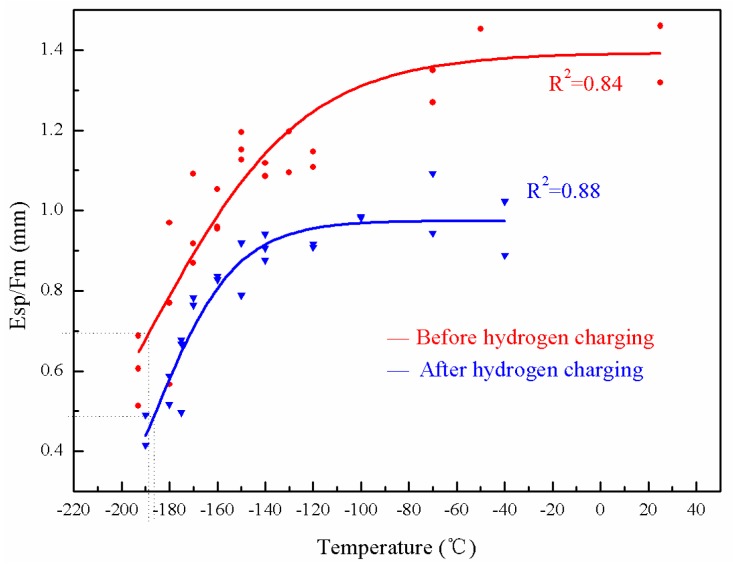
*T-E_sp_*/*F_m_* curve of the weld metal in de-embrittlement.

The temperature corresponding to 50% of the upper shelf value as *T_sp_* was defined above. The *T_sp_* of the weld metal in the as-received state and de-embrittled state were measured after hydrogen charging, as illustrated in Figure 6 and Figure 7.

Compared with Figure 6 and Figure 7, the upper shelf value of the weld metal in the as-received state and after de-embrittlement before hydrogen charging is higher. The charged specimen shows an obviously lower *E_sp_* compared with the uncharged specimen at the same temperatures, indicating the specimens become slightly more brittle after hydrogen charging. Table 5 shows more details concerning the differences between the weld metal in the de-embrittled state and the as-received state before and after hydrogen charging.

Comparing the data in Table 5, for both the as-received and the de-embrittled states, the *T_sp_* of the weld metal are almost the same.

### 3.5. Fractography of SPT

For further details about the fracture of the specimens after hydrogen charging and without hydrogen charging, the microstructures of the base metal and the weld metal were analyzed by scanning electron microscopy (SEM).

**Figure 8:**
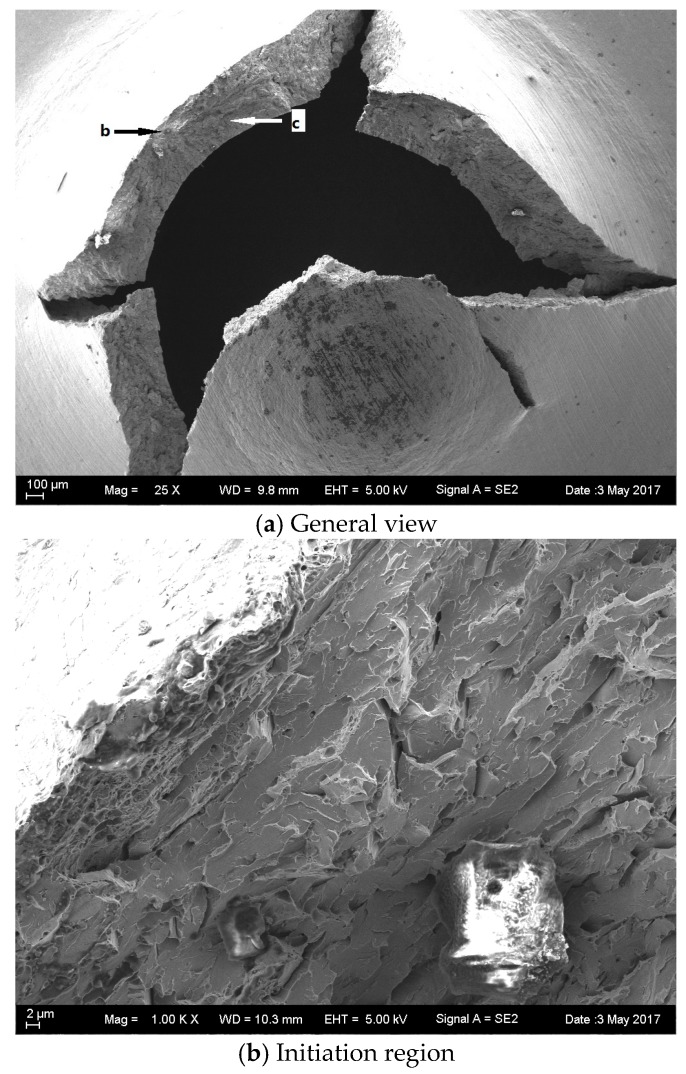

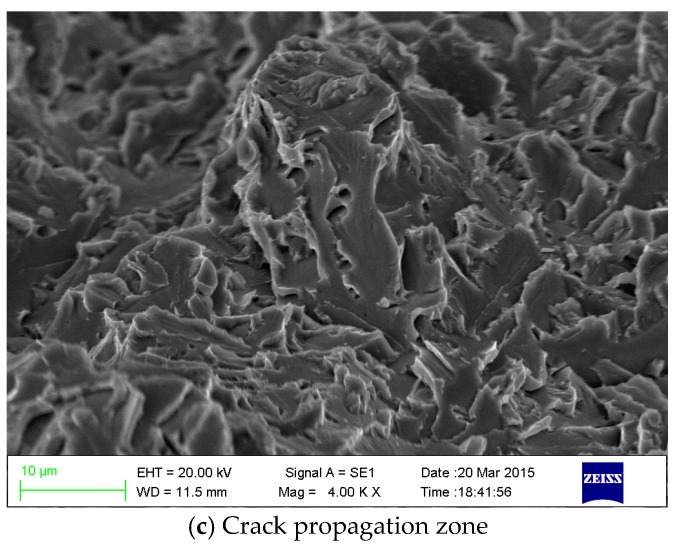
SEM images of the as-received base metal at −180 °C without hydrogen charging.

**Figure 9:**
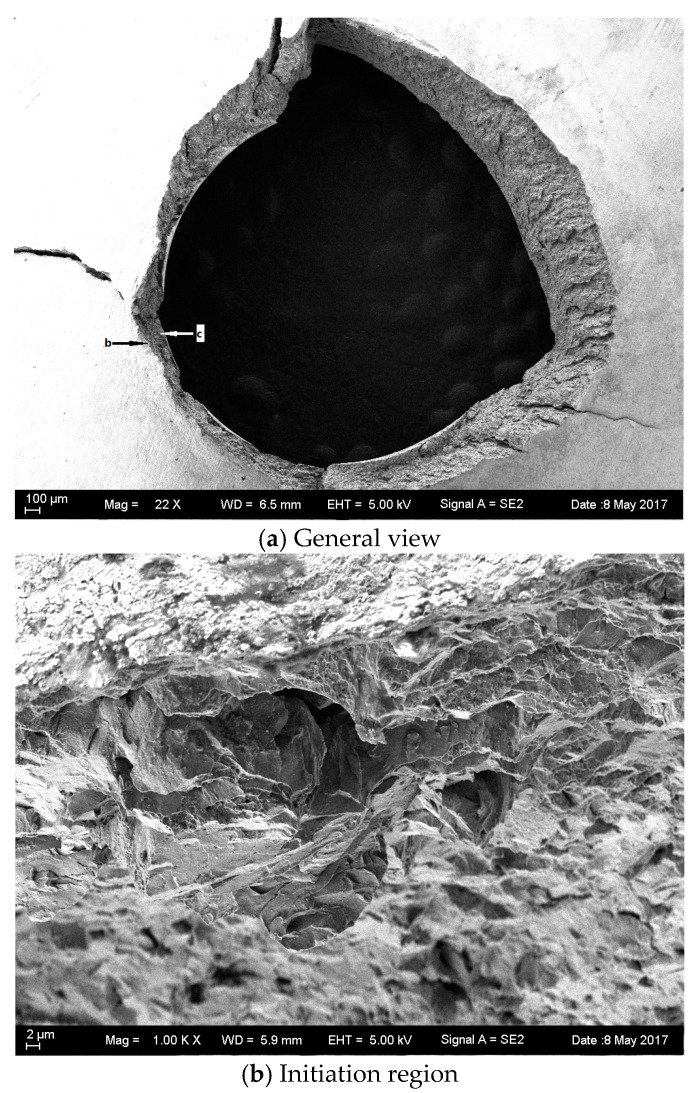

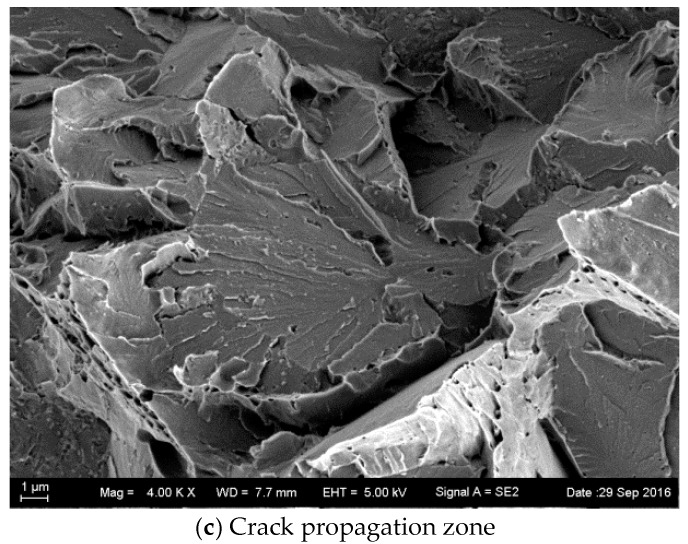
SEM images of the as-received base metal at −180 °C with hydrogen charging.

Figure 8 and Figure 9 show the SEM morphology of the as-received base specimen before and after hydrogen charging. A general birds-eye view of the crack profiles that occurred in the vicinity of the punch indentation on the small disc samples is shown in Figure 8a and Figure 9a. The cracking was circular in nature and confined to the actual punch indention. Fracture initiation occurred at the bottom surface location (opposite of the punch rod). Detailed views of the initiation area are shown in Figure 8b and Figure 9b. No actual initiation point was evident but it was significant that the outer reaches of the quasi-cleavage cracked. Figure 8c shows the quasi-cleavage fracture which is just the typical brittle fracture at low temperature. However, Figure 9c shows the typical characteristics of the HE fracture with crow’s feet morphology.

## 4. Discussion

### 4.1. Determination of T_sp_

There are some technical and theoretical issues for evaluating the energy transition temperature by using SPT; for example, the points of SPT fracture energy are rather scattered [20].

From the existing model, *T_sp_* is always derived from the Energy-Temperature Curve fitted with some special functions. Although, different functions are used in different laboratories, like the power function by Matocha [21], and the Boltzmann function in this paper. However, the maximum energy is difficult to determine, as shown in Figure 2, which leads to a larger error of *T_sp_*. When *E_sp_*/*F_m_* is used, this method leads to a constant value in the upper shelf (instead of the usual drop of *E_sp_* with rising temperature).

The results obtained indicate that *T_sp_* has the same physical meaning for FATT or vTr54. The lower shelf energy cannot be obtained for high-toughness steels. Within the liquid nitrogen temperature, the temperature corresponding to 50% of the upper shelf value is defined as the transition temperature of SPT. FATT is expressed as Equation (4).

Table 6 and Table 7 show the comparison of vTr54 between those values calculated by Equation (1) and those obtained by experiments in the as-received state. The data in Table 6 show that there is a relatively large difference between the vTr54 calculated using Equation (1) and the vTr54 obtained by experiments in the as-received state of the base metal. However, the difference between the data in Table 7 is small for the as-received state of the weld metal. This indicates that it is unreliable to define *T_sp_* by using 50% of upper shelf value for high toughness materials, whose *T_sp_* is near to or lower than the liquid nitrogen temperature.

The samples of SPT are disc-shaped test specimens without a notch. In ductile materials, the *T_sp_* is lower than the temperature of liquid nitrogen. Methods to improve the *T_sp_* of ductile materials led to the development of new disc-shaped specimens with a “U” notch in the axis of the disc plane [22].

### 4.2. Temper Embrittlement of 3Cr1Mo1/4V

Temper embrittlement occurs at temperatures below 600 °C, but mainly in the range from 350 to 550 °C [1]. Heavy wall Cr-Mo vessels fabricated over 30 years ago have demonstrated a high susceptibility to temper embrittlement after extended service at elevated temperatures. The threshold temperature where temper embrittlement is considered an issue by heavy wall vessel operators varies between 343 and 399 °C [23]. More recently the CrMo steels used in heavy wall reactors have lower levels of impurities with a very low *J-factor*, resulting in a significantly improved resistance to temper embrittlement. Significantly lower embrittlement indicated by lower shifts in the 54 J transition temperature occurs at temperatures below 399 °C in 2.25Cr1Mo and 2.25Cr1Mo 1/4V alloys that are widely used as hydrogenation reactor materials and their TE has been fully studied. Weld metal 2.25Cr1MoV with lower Cr and higher V content showed a superior resistance property to embrittlement as compared to conventional materials [24]. For these low *J-factor* (<100) steels, vTr54 is in general close to −100 °C for the base materials. Shifting due to temper embrittlement is not significant [1]. However, very little research was done for 3Cr1Mo1/4V steel due to rather infrequent use in the hydrogenation reactor. The results showed that a slight TE was found for service at 390 °C after ten years. For the weld metal, a 54 J impact energy is only about −30 °C for weld materials, which is very high compared with −100 °C for the base metal, and the TE cannot be neglected. This means that the users should pay attention to the temper embrittlement of the 3Cr1Mo1/4V after a long service period above 390 °C.

### 4.3. Hydrogen Embrittlement of 3Cr1Mo1/4V

The HE effect in hydrogenation reactor materials is very close to that attributed to TE, resulting in an upward shift of the transition temperature. The higher the hydrogen content, the higher the transition temperature. Another way to evaluate the effects of hydrogen is to consider the material fracture toughness at a given temperature and then, the higher the hydrogen content, the lower the material fracture toughness [1].

HE occurs as a result of hydrogen located within the bulk of the alloy during the application of a load. The embrittlement is manifested by the existence of non-ductile fracture modes, and reduced tensile strength. The most common HE mechanisms are the reduction in the lattice cohesive force (decohesion mechanism) and the hydrogen interaction with dislocations. Isolated areas are observed of either intergranular or transgranular fracture or interface separation, depending on the relative strength of the grain boundaries and other interfaces [25].

For an arbitrary but constant dissolved hydrogen content, the fracture mode changes, and there is the intergranular (IG) mode, quasi-cleavage (QC) mode, and microvoid coalescence (MVC) mode with a progressive increase in the stress intensity factor [26]. Cleavage fracture with special crow’s feet is a kind of typical fracture characteristic of HE [27] and this was clearly observed in Figure 9c, however, according to the *T_sp_* values before and after hydrogen charging, HE is not obvious.

In the case of steel-V which is the vanadium containing 2.25Cr-1Mo steel, the precipitated vanadium carbide is very effective for hydrogen trapping [28,29,30] and the major hydrogen trapping sites of steel-V are associated with the dispersion of fine precipitates such as vanadium carbide (VC). It is considered that the low susceptibility to HE of steel-V is linked to greater trapping and lower diffusivities due to the fine precipitation of V-rich carbides [26].

Figure 6 shows HE calculated based on TE and Figure 7 shows HE only. There is only a small difference between the curves in Figure 6 and Figure 7, which indicates that there was no significant indication of the synergetic action concerning the TE and HE. The Japan Pressure Vessel Research Committee (JPVRC, Tokyo, Japan) found that HE sensibility for the 2.25Cr-1Mo steel increases as the degree of temper embrittlement increases [29]. TE is not obvious and leads to no HE.

## 5. Conclusions

To evaluate the embrittlement of 3Cr1Mo1/4V hydrogenation reactor steel, small punch tests (SPT) were carried out for the as-received and de-embrittled materials which were cut from a test block exposed for ten years to 390 °C temperature in the reactor. The conclusions are as follows:

(1) *E_sp_* decreases with temperature increase at the upper shelf. However, it becomes constant at the upper shelf when *E_sp_* is divided by the maximum load. *E_sp_/F_m_*-Temperature is able to be used to better characterize the SPT energy transition.
(2) The SPT energy transition temperature (*T_sp_*) of the base metal and the weld metal increased 8.9 °C and 11.2 °C, respectively, in the de-embrittlement and as-received states before hydrogen charging, indicating that there is a slight extent of temper embrittlement for the 3Cr1Mo1/4V steel serviced at 390 °C for ten years.
(3) The specimen becomes slightly more brittle after hydrogen charging. The synergetic effect of temper embrittlement and hydrogen embrittlement has not been found in the 3Cr1Mo1/4V steel.

## Figures and Tables

**Table 1:** Mechanical properties of the test materials.

Yield Strength *R_el_* (MPa)	Maximum Strength *R_m_* (MPa)	Percentage of Elongation after Fracture *δ*_4_ (%)	Ductile-Brittle Transition Temperature vTr54 (°C)
466	590	28	−71.1

**Table 2:** Chemical composition of the test materials (wt %).

Elements	C	Si	Mn	P	S	Cr	Mo	Ni	Cu	Sb	Sn	As
Base material	0.115	0.07	0.52	0.006	0.006	3.03	0.93	0.1	0.082	0.008	0.0078	0.0067
Weld material	0.074	0.155	1.057	0.006	0.005	2.862	0.904	0.045	0.09	0.0073	0.0039	0.0051

**Table 3:** *F_m_* and *U_f_* at different temperatures of the weld metal.

Temperature (°C)	*F_m_* (N)	*U_f_* (mm)
−20	1861.08	2.023
−120	2392.38	1.945
−150	2765.74	1.917
−160	2700.46	1.747
−180	2628.22	1.509
−190	1369.10	0.713

**Table 4:** Small punch test and Charpy impact test results.

Material	As-Received State vTr54	De-Embrittlement vTr54	As-Received State *T_sp_*	De-Embrittlement *T_sp_*	ΔvTr54	Δ*T_sp_*
Base metal (°C)	−60.3	−71	−181	−189.9	10.7	8.9
Weld metal (°C)	−17.3	−31	−176.9	−188.1	13.7	11.2

**Table 5:** *T_sp_* of the weld metal in different conditions.

State	Before Hydrogen Charging	After Hydrogen Charging	Δ*T_sp_*
*T_sp_* of As-received state (°C)	−176.9	−174.8	2.1
*T_sp_* of De-embrittlement (°C)	−188.1	−186.4	1.7

**Table 6:** Comparison of vTr54 in the as-received state of the base metal.

State	*T_sp_*	vTr54 by Equations (3) and (4)	vTr54 (Tests)	ΔvTr54
As-received (°C)	−181.0	−22.7	−60.3	37.6
De-embrittlement (°C)	−189.9	−36.7	−71.0	34.3

**Table 7:** Comparison of vTr54 in the as-received state of the weld metal.

State	*T_sp_*	vTr54 by Equations (3) and (4)	vTr54 (Tests)	ΔvTr54
As-received (°C)	−176.9	−16.2	−17.3	1.1
De-embrittlement (°C)	−188.1	−33.9	−31	−2.9

## References

[1] Pillot S., Chauvy C., Corre S., Coudreuse L., Gingell A., Héritier D. (2013). Effect of temper and hydrogen embrittlement on mechanical properties of 2.25Cr–1Mo steel grades—Application to minimum pressurizing temperature (mpt) issues. Part I: General considerations & materials’ properties. Int. J. Press. Vessel. Pip..

[2] Zabil’Skii V.V. (1987). Temper embrittlement of structural alloy steels (review). Met. Sci. Heat Treat..

[3] Zhang X., Zhou C. Study on the Hydrogen Effect on the Temper Embrittlement of 2.25Cr-1Mo Steel. Proceedings of the International Conference on Nuclear Engineering.

[4] Bulloch J.H., Crowe D. (1998). Embrittlement observed in Cr–Mo turbine bolts after service. Theor. Appl. Fract. Mech..

[5] Pillot S., Chauvy C., Corre S., Coudreuse L., Gingell A., Héritier D. (2013). Effect of temper and hydrogen embrittlement on mechanical properties of 2.25Cr–1Mo steel grades—Application to minimum pressurizing temperature (MPT) issues. Part II: Vintage reactors & MPT determination. Int. J. Press. Vessel. Pip..

[6] Guan K.S., Wang Z.W. (2011). Effects of material microdefects on results of small punch test. Mater. Mech. Eng..

[7] Manahan M.P., Argon A.S., Harling O.K. (1981). The development of a miniaturized disk bend test for the determination of postirradiation mechanical properties. J. Nucl. Mater..

[8] Chang Y.S., Kim J.M., Choi J.B., Kim Y.J., Kim M.C., Lee B.S. (2008). Derivation of ductile fracture resistance by use of small punch specimens. Eng. Fract. Mech..

[9] Mang A.I., Zhen Y., ZhiWen W. (2002). Origination, development and application of small punch test method. J. Mech. Strength.

[10] Matocha K., Filip M., Karthik V., Kumar R.V., Lacalle R., Tonti A., Matocha K., Hurst R, Sun W. (2012). Results of the Round Robin Test for Determination of TSP of P22 Steel by Small Punch Tests. Determination of Mechanical Properties by Small Punch and Other Miniature Testing Techniques, Proceedings of the 2nd International Conference on SSTT, Ostrava, Czech Republic, October 2012.

[11] Prescott G.R. (1998). Operating Issues of Aging Reactors.

[12] Hurst R., Matocha K. (2012). Where are we now with the European Code of Practice for Small Punch Testing. Determination of Mechanical Properties of Materials by Small Punch and Other Miniature Testing Techniques.

[13] GB/T 29459-2012 (2012). Small Punch Test Methods of Metallic Materials for In-Service Pressure Equipments. Part 1 and Part 2.

[14] Guan K., Hua L., Wang Q., Zou X., Song M. (2011). Assessment of toughness in long term service CrMo low alloy steel by fracture toughness and small punch test. Nucl. Eng. Des..

[15] (2008). Metallic Materials-Charpy Pendulum Impact Test Method.

[16] (2008). Impact Test Methods on Welded Joints.

[17] Yang J., Guan K. (2014). Evaluation of Hydrogen Embrittlement Susceptibility of 2205 Duplex Stainless Weld Joint.

[18] Albert S.K., Padhy G.K. (2014). A Brief Review on Methods for Diffusible Hydrogen Measurement in Welds. Indian Weld. J..

[19] Finarelli D., Carsughi F., Jung P. (2008). The small ball punch test at FZJ. J. Nucl. Mater..

[20] Zhang E.Y., Yu C., Xu Y.-F., Guan K.S. (2014). Effects of temper embrittlement on mechanical properties of hydrogenation reactor steel weld determined by small punch test. Mater. Mech. Eng..

[21] Matocha K. (2009). The evaluation of materials properties of inservice components by small punch tests. J. KONES.

[22] Matocha K. (2014). The Use of Small Punch Tests for Determination of Fracture behaviour of Ferritic Steels. Procedia Eng..

[23] (2007). API 934-F. Guidance on Heavy Wall Reactor Startup and Shutdown.

[24] Taniguchi G., Yamashita K., Otsu M., Nako H., Sakata M. (2015). A study on the development of creep rupture and temper embrittlement properties in 21/4Cr-1Mo-V steel weld metal. Weld. World.

[25] Eliaz N., Shachar A., Tal B., Eliezer D. (2002). Characteristics of hydrogen embrittlement, stress corrosion cracking and tempered martensite embrittlement in high-strength steels. Eng. Fail. Anal..

[26] Shimazu H., Konosu S., Tanaka Y., Yuga M., Yamamoto H., Ohtsuka N. Combined Effect of Temper and Hydrogen Embrittlement on Threshold for Hydrogen–Induced Fracture in Cr-Mo Steels. Proceedings of the ASME 2012 Pressure Vessels and Piping Conference.

[27] Woodtli J., Kieselbach R. (2000). Damage due to hydrogen embrittlement and stress corrosion cracking. Eng. Fail. Anal..

[28] Masaoka I., Kinoshita K., Chiba R. (1985). Hydrogen Attack Limit of 2.25Cr-1Mo Steel.

[29] JPVRC (1985). Embrittlement of Pressure Vessel Steels in High Temperature, High Pressure Hydrogen Environment.

[30] JPVRC (1985). Hydrogen Embrittlement of Bond Structure between Stainless Steel Overlay and Base Metal.

